# Holistic and featural processing’s link to face recognition varies by individual and task

**DOI:** 10.1038/s41598-023-44164-w

**Published:** 2023-10-06

**Authors:** Bryan Qi Zheng Leong, Alejandro J. Estudillo, Ahamed Miflah Hussain Ismail

**Affiliations:** 1 School of Psychology, University of Nottingham Malaysia, Semenyih, Malaysia; 2 Department of Psychology, Bournemouth University, Poole House Talbot Campus, Poole, BH12 5BB UK

**Keywords:** Human behaviour, Long-term memory

## Abstract

While it is generally accepted that holistic processing facilitates face recognition, recent studies suggest that poor recognition might also arise from imprecise perception of local features in the face. This study aimed to examine to what extent holistic and featural processing relates to individual differences in face recognition ability (FRA), during face learning (Experiment 1) and face recognition (Experiment 2). Participants performed two tasks: (1) The “Cambridge Face Memory Test-Chinese” which measured participants’ FRAs, and (2) an “old/new recognition memory test” encompassing whole faces (preserving holistic and featural processing) and faces revealed through a dynamic aperture (impairing holistic processing but preserving featural processing). Our results showed that participants recognised faces more accurately in conditions when holistic information was preserved, than when it is impaired. We also show that the better use of holistic processing during face learning and face recognition was associated with better FRAs. However, enhanced featural processing during recognition, but not during learning, was related to better FRAs. Together, our findings demonstrate that good face recognition depends on distinct roles played by holistic and featural processing at different stages of face recognition.

## Introduction

Recognising the identity of an individual by perceiving their face is a fundamental social skill. Most human faces adhere to a standard template and configuration of facial features such as the eyes, nose, and mouth. While the isolated processing of different facial features is known as “featural processing”, the combination of these facial features and their configuration into a whole is referred to as “holistic processing”^1^. Although both processes are believed to contribute to face recognition, the popular view is that holistic processing is relatively more crucial^2–4^. However, the contribution of holistic and featural processing to different stages of the face recognition process (i.e., learning vs. recognition) and their relationship with individual differences in face recognition are largely unknown. This study aims to shed light on these questions.

In typical adults, the *face inversion*, *composite face* and *part-whole* tasks are conventionally used to demonstrate the dominance of holistic processing in face recognition^5,6^. In the inversion effect, recognition is more accurate for *upright* (experimental condition) faces than for *inverted* faces (control condition), since the latter impairs holistic processing^3,7,8^. In the composite effect^4,9,10^, when the top half of one identity’s face is spatially *aligned* with the bottom half of another identity (experimental condition), the two halves are fused to create an illusory identity, and this impairs recognising the source identity of each half. However, this impairment disappears when the two halves are *misaligned* (control condition) and holistic processing is disrupted. In the part-whole effect^11–13^, recognising an individual part (e.g., a nose) of a previously learnt face is more accurate when it is presented in the context of a *whole* face (experimental condition) rather than an isolated *part* (control condition). Face parts are believed to be encoded by engaging holistic processes that integrate them into a whole, and therefore part recognition is best when the same processes can be engaged during recognition (i.e., whole condition). Interestingly, some studies have reported positive correlations between these indexes and face identification^14–16^, pointing to holistic processing as the underlying mechanism explaining individual differences in face recognition (but see Konar et al.^17^; Verhallen et al.^18^).

However, there is also emerging evidence suggesting that featural processing is important for face identification too. For instance, Cabeza and Kato^19^ found that participants were equally prone to falsely recognise novel faces (what they called “prototype faces”) that only had either holistic information or featural information preserved from previously learnt faces. This reflects that both holistic and featural information were encoded and stored, and that they may be equally important in face recognition. More recently, DeGutis et al.^14^ used the part-whole and composite tasks to demonstrate that both holistic and featural processing contributes independently and significantly to face recognition abilities (FRA). First, they obtained an independent measure of recognition based on featural processing by calculating the accuracy for the control conditions (e.g., part condition in the part-whole task) where holistic information is disrupted. Second, they regressed the variance of the control conditions from the experimental conditions (e.g., whole condition in the part-whole task) to obtain an independent score of holistic processing. They found significant positive correlations between these independent estimates of holistic as well as featural processing and their measures of FRA (scores in the Cambridge Face Memory Test; CFMT^20^). Furthermore, it has also been suggested that featural processing is more important for the recognition of unfamiliar faces than familiar faces^21^. For example, Lobmaier and Mast^22^ found that matching two sequentially presented faces is relatively more impaired when the two faces are blurred (i.e., to disrupt featural processing) than when they are scrambled (i.e., to disrupt holistic processing), but this disadvantage for blurred faces was more pronounced for novel faces than previously learnt faces.

With conventional measures of holistic processing (i.e., composite, part-whole and inversion effects), the assumption is that their experimental manipulations (e.g., misaligning faces in the composite task) are meant to disrupt holistic processing. However, these measures are not free of criticism as there are secondary factors that could drive the same effects too^23^. For example, in the part-whole task, faces are always encoded in their whole, arguing that the part-whole effect could be driven by encoding specificity^24^. Further, the experimental condition generally contains more facial information than the control condition. Here, the so-called holistic advantage measured by the part-whole effect could reflect differences in the amount of featural information contained between the two conditions. Recent studies have also criticised the functional significance of the composite face task^6,25^. For instance, Fitousi^25^ showed that aligned composite faces (that are often used to demonstrate interference from holistic processing) were not affected by the Garner interference paradigm. In other words, participants were perfectly capable of selectively attending to target facial features even when other irrelevant features were manipulated, casting doubt on the fact that holistic processing may be interfering with perception in aligned composites. To control for secondary cognitive factors, studies have often adopted these two holistic measures with the inversion effect. Following this argument, the pure contribution of holistic processing would be observed when the part-whole and composite effects are only present with upright faces and disappear for inverted faces^23^.

With regard to the inversion task, the most common interpretation is that the upright condition facilitates holistic processing^3,8^. If that is the case, when observers are forced to view both upright and inverted faces in a featural manner, the inversion effect should be reduced, or disappear. Murphy and Cook^26^ used the fixed-trajectory aperture paradigm (FTAP) to examine this hypothesis. This paradigm has two conditions: (1) the “whole” condition in which the entire face is visible to the observer, and (2) the “aperture” condition in which a transparent, rectangular window smoothly moves from the top of the face to the bottom, revealing parts of the face in a sequential order. Murphy and Cook^26^ found that faces are recognised better in the whole conditions compared to the aperture conditions (i.e., the “aperture effect”), suggesting that the dynamic aperture successfully disrupts holistic processing. Interestingly, the magnitude of the inversion effect (i.e., the difference between the upright condition and the inverted condition) was comparable in both the whole and aperture conditions (see also Murphy and Cook^27^). This is in stark contrast with the holistic accounts of the face inversion effect, which predicts that an inversion effect should only be observed when the entire face is fully visible.

Therefore, Murphy and Cook’s findings challenge the view that the inversion effect disrupts only holistic processing, at the same time providing a paradigm that systematically disrupts or facilitates holistic processing. Interestingly, the FTAP is also a good paradigm to measure individual differences in holistic and featural processing. For example, Tsantani et al.^28^ showed that Developmental Prosopagnosics (DPs) are less accurate in recognising upright faces in both the whole and the aperture conditions, compared to typical adults without face recognition deficits. However, the magnitude of the holistic advantage (i.e., higher accuracy in the whole compared to the aperture condition) was similar between DPs and typical adults. This shows that DPs are impaired in processing faces featurally but not holistically.

### Learning and recognition

Recognising the identity of an unfamiliar face is a product of at least two exposures to the same face. In its simplest order, the first exposure results in the observer learning the identity of the face and during the second exposure, the observer recognises a face they have learnt before. The distinction between these two stages is supported by neuroimaging evidence that showed different brain regions were involved during the learning and recognition of faces^29^. Interestingly, most studies attempting to examine the contribution of holistic and featural processing to face identification do not specifically address the role of these processes in the learning and recognition of faces.

Some studies have used oculomotor behaviour to index the processes involved during visual sampling^30^. Measuring fixations, Henderson et al.^31^ found that face recognition is better if observers were allowed to freely fixate on the face during learning, rather than being forced to learn faces with just a single fixation. Further, eye movement patterns during recognition were comparable between conditions in which participants learnt faces by freely fixating them and by means of a single fixation. These findings suggest that, although recognition ability depends on how observers sampled facial information during learning, the information sampling strategy employed by observers during recognition is independent of how faces were learnt. Henderson et al.^31^ also reported that when observers freely fixated on faces during learning and recognition, they were largely directed at internal facial features. Although these fixations were attributed to processing holistic information, we could also assume that they served a simpler purpose of separately encoding individual features at high resolution, in other words, featural processing^32,33^. Lastly, Henderson et al. also reported that when observers were allowed to freely explore faces, fixations during recognition were much more restricted than those during learning. This could suggest greater reliance on featural processing during learning and/or greater reliance on holistic processing during recognition. While both interpretations are possible, there is no way to be certain of the purpose of fixations, as they can be used, at the best, as indirect measures of these processes^33^.

A recent study by Dunn et al.^34^, using a gaze-contingent paradigm, further examined the contributions of both holistic and featural processing in face recognition at the learning and recognition stages. Faces were viewed either in full-view or through circular apertures varying in sizes. When observers were allowed to sample faces freely during face learning and face recognition, super-recognizers (SRs) had a broader gaze distribution and more exploratory fixations than control participants. Most importantly, SRs were consistently better than control participants regardless of the aperture size. This indicates that the underlying perceptual processes contributing to superior face recognition can be explained by featural processing. Interestingly, these differences were more evident during face learning than during face recognition. In line with Henderson et al.^31^, these findings suggest that broader exploration of the face during face learning facilitates face recognition and could quantitatively explain individual differences in face recognition.

### The present study

To explore the contribution of holistic and featural processing at learning and recognition and their relationship with individual differences in FRA, the present study uses the FTAP in each stage separately. In Experiment 1, to isolate the contribution of holistic and featural processing during learning, faces were learned either through an aperture or in full-view. However, during the recognition stage, all faces were viewed in full-view. In Experiment 2, all faces were viewed in their entirety during learning. However, during the recognition stage, some faces were viewed through an aperture while others were viewed in their entirety. This allowed us to isolate the contribution of holistic and featural processing during the recognition stage to FRA. In addition, to measure individual differences in face recognition, observers performed the Cambridge Face Memory Test-Chinese (CFMT-Chi)^35^, a highly reliable and valid measure of individual differences in face recognition skills^36^.

## Results

**Figure 1:**
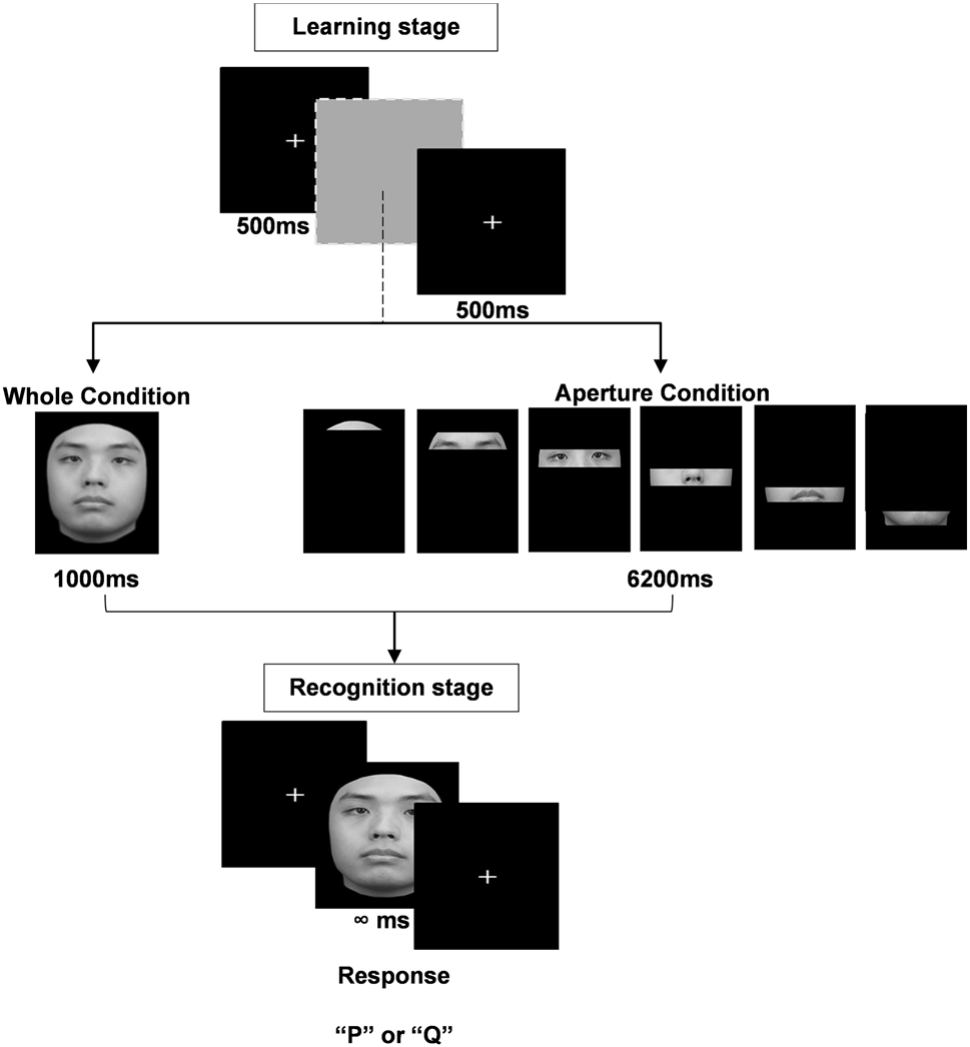
Chronological procedure and examples of stimuli in the old/new recognition memory task used in Experiment 1. In the aperture condition (centre right), a dynamic window moves smoothly across the face image from top to bottom (images from left to right).

Holistic and featural processing abilities were assessed with the FTAP in an old/new recognition memory task (RMT). The task involved two stages as shown in Fig. 1: a “learning” stage where participants learn a series of faces, and a “recognition” stage, where participants attempt to recognise the learnt face among a set of faces that contains new faces too. In Experiment 1, faces in the learning stage were presented in their entirety (“whole condition”) or through the fixed-trajectory aperture (“aperture condition”). Faces in the recognition stage were always presented in their entirety for both conditions, thus, scores here were always computed from the recognition of full faces. This manipulation was reversed in Experiment 2. Learning stage faces were always presented in their entirety, whereas recognition stage faces were shown in their entirety or through the aperture. Hence, scores here were computed from the recognition of either full or aperture faces. Briefly, recognition performance in the aperture condition of the RMT informs us how good our participants are with featural processing. The improvement in performance in the whole condition compared to the aperture condition of the RMT is a measure of the magnitude of the holistic advantage experienced by participants, i.e., how good they were with holistic processing. To obtain a standardised measure of FRA, we used the CFMT-Chi^35^. Correlating the aperture condition’s accuracy and the holistic advantage calculated from the old/new recognition task to the CFMT-Chi would tell us to what extent featural and holistic processing relates to FRA, respectively.

**Figure 2:**
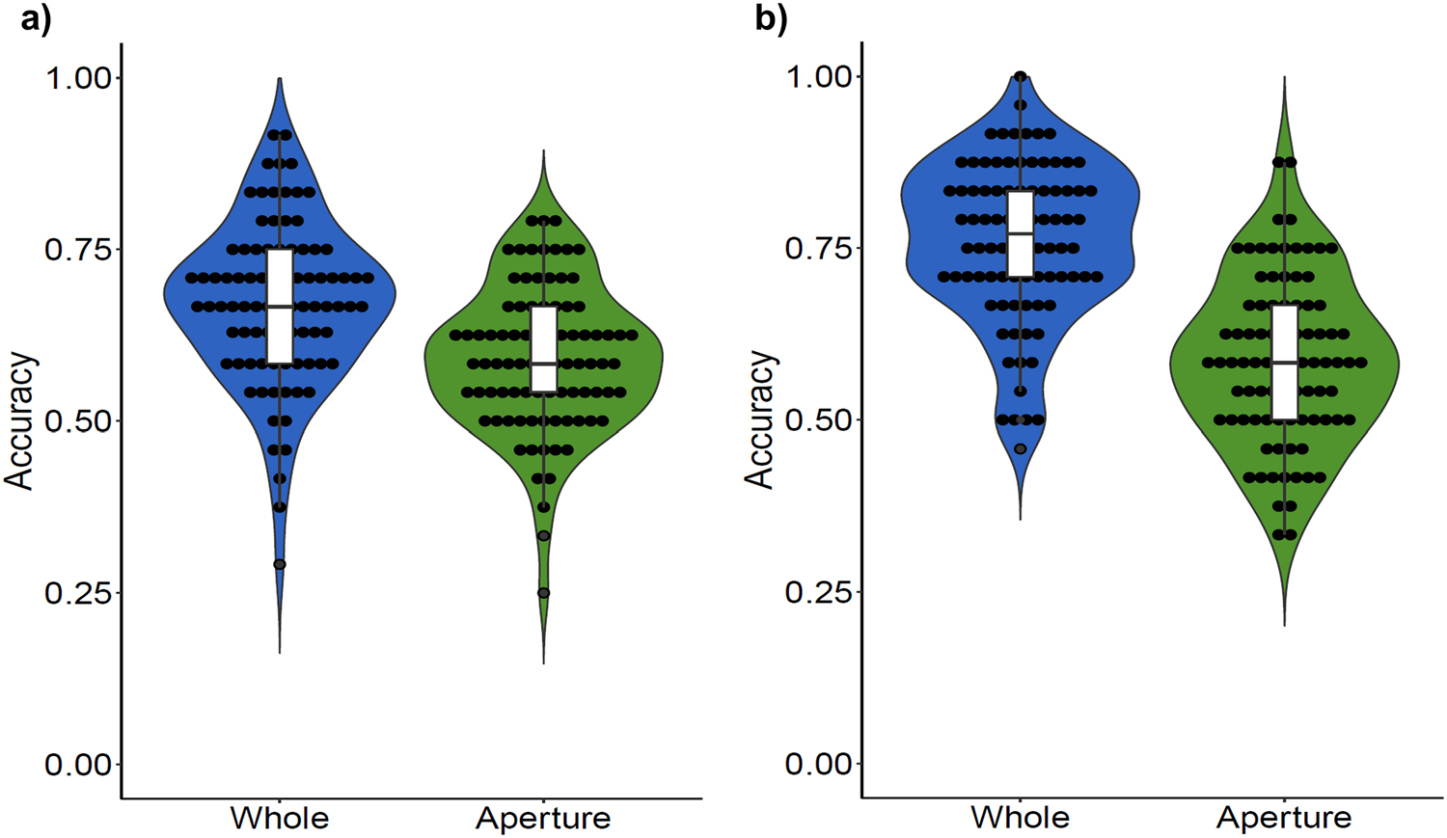
Mean accuracies for the whole (blue) and aperture (green) conditions from (**a**) Experiment 1 and (**b**) Experiment 2. Black-filled circles represent accuracy scores from individual participants.

The maximum achievable score (e.g., sum of correct responses) for the CFMT-Chi is 72, in which our current sample had a mean score of 57.98 (*SD* = 8.93) in Experiment 1 and 58.28 (*SD* = 8.53) in Experiment 2. As revealed by a two-tailed independent-samples *t*-test, the mean CFMT-Chi scores for both experiments were not significantly different from each other, *t*(171) = − 0.23, *p* = 0.820, *η*_*p*_^2^ = − 0.035. This shows that our participants’ FRA are largely similar between the two experiments, as well as with those of previous studies^35–39^. Mean accuracy scores of the RMT were calculated separately for each of the two viewing conditions: “whole” and “aperture” (Fig. 2). Two-tailed paired samples *t*-tests were conducted to compare accuracy scores between the two conditions of the RMT. In Experiment 1, there was a significant difference in the mean scores between the conditions, *t*(86) = 5.67, *p* < 0.001, *η*_*p*_^2^ = 0.607, in which mean accuracy in the whole condition (*M* = 0.672, *SD* = 0.117) was significantly higher than that of the aperture condition (*M* = 0.590, *SD* = 0.104). Similarly, in Experiment 2, we found that there was a significant difference in accuracy between the two conditions, *t*(85) = 11.21, *p* < 0.001, *η*_*p*_^2^ = 1.209, in which mean accuracy for the whole condition (*M* = 0.759, *SD* = 0.116) was higher than that of the aperture condition (*M* = 0.586, *SD* = 0.120). In both experiments, one-sample *t*-tests revealed that the accuracy in the aperture conditions were significantly better than chance (accuracy more than 0.5) at the group level: *t*(86) = 7.978, *p* < 0.001 (for Experiment 1) and *t*(85) = 6.638, *p* < 0.001 (for Experiment 2). A further independent-samples *t*-test confirmed that these mean accuracies are comparable between experiments, *t*(172) = 0.222, *p* = 0.824.

Traditionally, the holistic advantage has been calculated using subtraction methods^6^. In the case of the FTAP, this method would involve subtracting the mean accuracy in the aperture condition from the mean accuracy in the whole condition. However, subtraction methods can be difficult to interpret^14^, as a lower value for the aperture effect can indicate close to ceiling performance in the aperture condition, close to floor performance in the whole condition, or both. Thus, in the present study, we used the “regression” method^6,14^ to calculate the holistic advantage experienced by participants in the whole condition, after accounting for the variation in performance that the whole condition shares with the aperture condition. Using the equation of the line of best fit of the overall scores, each participant’s expected score on the whole condition (i.e., residual scores) was calculated based on their performance in the aperture condition. Here, accuracy in the aperture condition is regressed from the whole condition to compute residuals, which we termed “residuals of aperture effect” (RAE). A higher RAE score indicates stronger holistic processing.

**Figure 3:**
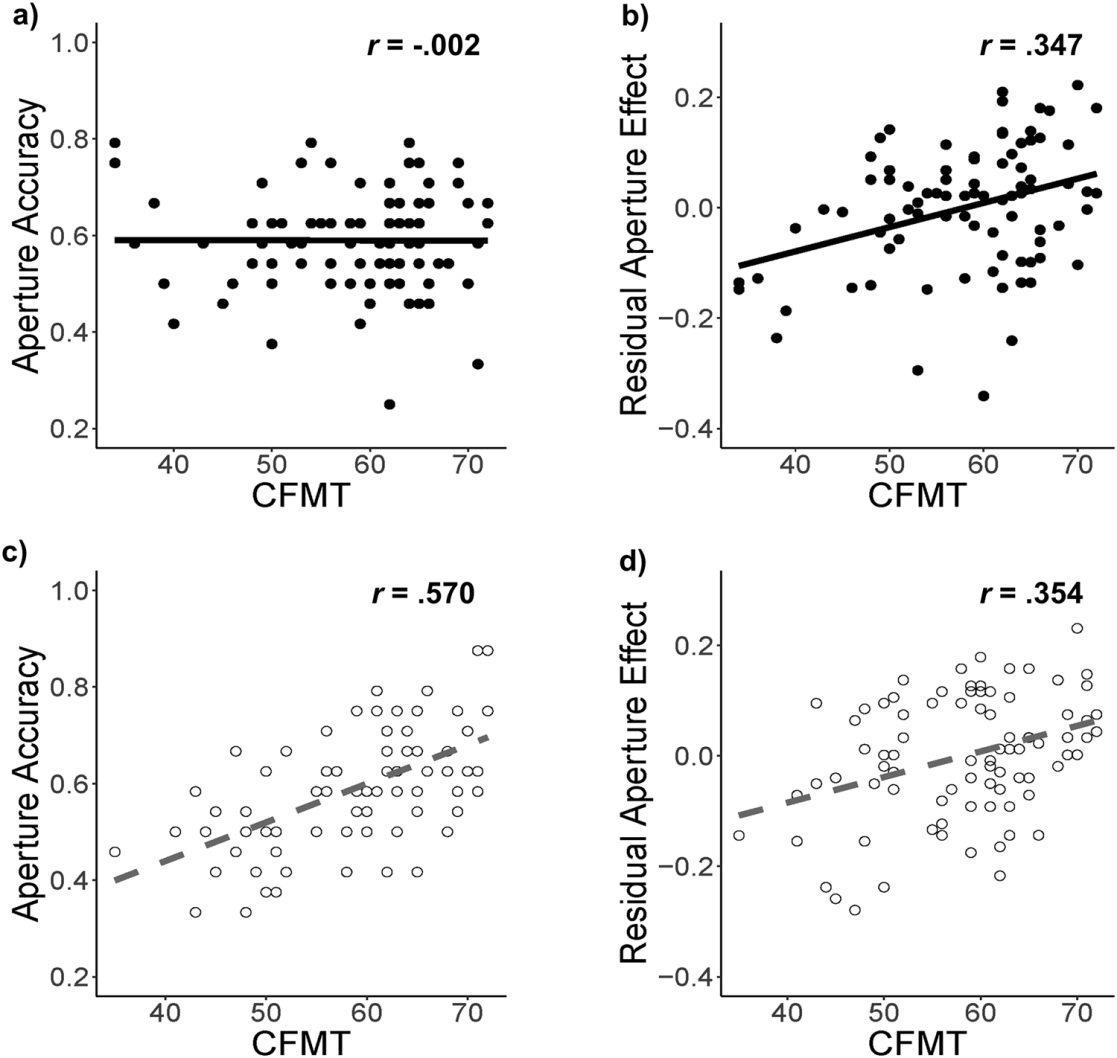
Correlation analyses from Experiments 1 (black) and 2 (grey). Black circles and grey annulus represent scores from individual participants in Experiment 1 and Experiment 2, respectively. Black solid lines and grey dashed lines are least-squares regression fits to individual data from Experiments 1 and 2, respectively.

Next, we ran a number of Pearson’s product-moment correlation tests for data obtained from Experiment 1. First, to explore if both tasks are measuring similar constructs, we correlated the accuracies of the whole condition in the RMT with the CFMT-Chi scores. The test showed a significant positive correlation between the two tasks, *r*(85) = 0.334, *p* = 0.002. Second, to explore the relationship between featural processing ability and FRA, we correlated the accuracies of the aperture condition with the CFMT-Chi scores, and the test showed no significant correlation between the two, *r*(85) = − 0.002, *p* = 0.986 (Fig. 3a). Third, to explore the relationship between holistic processing ability and FRA, we correlated measures of holistic advantage with CFMT-Chi scores. There was a significant positive correlation between the RAE scores and CFMT-Chi scores (Fig. 3b), *r*(85) = 0.347, *p* < 0.001. For Experiment 2, we found a positive correlation between the accuracy in the whole condition and their respective scores on the CFMT-Chi, (*r*(84) = 0.489, *p* < 0.001). There was also a strong positive correlation between the accuracy in the aperture condition and their respective scores on the CFMT-Chi (Fig. 3c), (*r*(84) = 0.570, *p* < 0.001). Particularly, the higher the participants’ FRA, the more accurate they were in the “aperture” condition. Additionally, there was a significant positive correlation between the RAE and CFMT-Chi scores (Fig. 3d), *r*(84) = 0.354, *p* < 0.001. Since some participants were performing at (or close to) chance level in our experimental conditions (especially for the aperture conditions of both experiments), it is possible that floor effects can account for some correlations (or the lack of it). To address this, we also correlated whole accuracy, aperture accuracy and RAE scores with CFMT-Chi scores after excluding participants who did not score above chance, as identified by binomial probability tests. Importantly, the pattern of results remained the same (see Online Supplementary Material).

To compare the strengths of correlations between the two experiments and between the whole and aperture conditions within each experiment, we transformed the Pearson’s correlation coefficient values into *z* scores (i.e., Fisher’s *r* to *z* transformation)^40^. We found a significant difference in coefficients between Experiment 1 and 2 for the correlations between aperture accuracy and CFMT-Chi (*z* = − 4.197, *p* < 0.001), but not for the correlations between RAE and CFMT-Chi (*z* = − 0.052, *p* = 0.479). Specifically, the correlation coefficient between aperture accuracy and CFMT-Chi was larger in Experiment 2 than in Experiment 1. Additionally, the correlation coefficients between aperture accuracy with CFMT-Chi, and RAE with CFMT-Chi, were significantly different in Experiment 1 (*z* = − 2.359, *p* = 0.009) and Experiment 2 (*z* = 1.788, *p* = 0.037). Particularly, the correlation with CFMT-Chi was stronger for RAE (i.e., holistic processing) in Experiment 1, but the correlation with CFMT-Chi was stronger for aperture accuracy (i.e., featural processing) in Experiment 2. Lastly, for the correlations between whole condition’s accuracy and CFMT-Chi scores, the coefficients were comparable between Experiments 1 and 2 (*z* = − 1.211, *p* = 0.113).

## Discussion

The purpose of the present study was to examine the role of holistic and featural processing in face recognition ability (FRA). Both experiments showed that forcing observers to rely on featural processing with a small aperture reduced recognition accuracy significantly. This impairment was observed irrespective of whether the aperture was applied during face learning or recognition. One unique characteristic of our study is that we measured to what extent featural and holistic processing can explain FRA at different stages of face recognition, separately. In Experiment 1, we found that accuracy for recognising faces learnt through featural processing was uniform, *albeit* poor, across the whole spectrum of FRA. To our knowledge, no past study had systematically restricted participants along the FRA spectrum to featural processing during face learning. Accordingly, our findings are novel in isolating the contribution of featural processing during face learning to face recognition ability. Based on our findings, featural processing during face learning does not account for individual differences in face recognition abilities.

In Experiment 2, we found that individuals with better FRA were also better at using featural processing during recognition than individuals with poor FRA. This suggests that featural processing during face recognition contributes to identifying learnt faces, and it is in support of past findings showing that good recognisers make good use of featural processing when attempting to recognise a learnt face. These past studies have used various tasks to assess the contribution of featural processing (e.g., part-whole task, familiar face recognition test) in recognising famous faces as well as recently learnt unfamiliar faces^28,41,42^. Nonetheless, there are also some exceptions^34,43^. One could argue that the lack of correlation between featural processing ability and FRA in Experiment 1 is a result of floor effects. However, accuracies were comparable across both experiments and above chance. In addition, individual differences in the aperture condition were related to FRA in Experiment 2, but not in Experiment 1. Therefore, floor effects are unlikely to explain a lack of correlation in Experiment 1.

In line with Dunn et al.^34^, we found that featural processing is positively associated with FRA. However, we only found this correlation during face recognition and not face learning (i.e., Experiment 1). These disparities could be the result of our viewing manipulations. Dunn et al. allowed observers to actively explore the faces, whereas the FTAP constraints all observers to learn faces in a similar fashion, which could interfere with unique perceptual encoding strategies used by good recognizers. For instance, Dunn et al. found that super recognisers (SRs) had broader gaze distributions and more fixations than typical observers, but these differences were more apparent during face learning. In contrast, Abudarham et al.^43^ showed that Developmental Prosopagnosics (DPs) and SRs are similarly good at featural processing. However, DPs tend to be heterogenous in deficits, with some cases having featural processing deficits and some not^44,45^, and deficits can be qualitatively different from neurotypicals with poor FRA (e.g., atypical sampling of faces)^46–48^.

Additionally, our RAE scores showed that people’s ability to process faces holistically (but not featurally) during face learning could be a strong determinant of their FRA. The relationship found in Experiment 2 further supports previous findings showing that higher face recognition abilities are associated with stronger holistic processing^14–16^. Nonetheless, as we found, why would good recognisers rely more on processing holistic but not featural representations of a face during face learning? We encounter a large number of faces in everyday life. Obviously, the more faces we can store in our memory, the better our social interactions would be. However, storing individual features of every single face we encounter would be very taxing for human memory. Holistic representations provide a way of reducing this memory load, by allowing us to store more identities in the form of a simplified gist (see Curby and Gauthier^49^; Pertzov et al.^50^). Moreover, holistic information of faces is more stable in memory than featural information^4,7,51^. For example, Peters and Kemner^52^ showed that long-term memory for faces is better when face identities were learnt from their low spatial frequencies conveying holistic information than from their high spatial frequencies conveying fine details of features. Given that holistic representations allow us to efficiently utilise memory and form stable traces over time, it would be expected that good recognisers make better use of holistic processing than featural processing during face learning.

Why would good recognisers rely on both holistic and featural processing during recognition, but not face learning? Some studies have demonstrated that when we attempt to recognise a face, we follow a course-to-fine strategy^53–55^. Here, a holistic representation of the to-be-recognised face is initially matched to face representations in our memory to narrow down the most likely candidate representations^53^. Next, in an empirical sense, features of the to-be-recognised face are compared with those selected representations in memory, whereby identity-specific, distinct features could help to distinguish a learnt identity from other similar-resembling faces. Extending this explanation to our case, it appears important that we compare a to-be-recognised face to memory representations both at the holistic and featural levels, and good recognisers might be adept at doing both.

We would also like to emphasise on an interesting finding of our study. In the aperture condition of Experiment 1, when participants’ face learning was restricted to featural processing, even good recognisers failed to use this information. However, in Experiment 2, when we allowed participants to learn faces freely (i.e., not restricting the processing), good recognisers were able to recognise these faces better even when holistic processing was largely interrupted during recognition due to the aperture. As the FRA of participants decreased, this advantage with featural information diminished. Based on this, we can claim that forming a holistic representation when learning a face is also important for good recognisers to effectively use featural information during recognition. If that’s the case, a weak holistic representation formed by poor recognisers during learning may have led to poor use of both holistic and featural information during recognition (as shown in Experiment 2; Fig. 3).

However, our study is not without limitations. First, we did not account for congruency effects between face learning and face recognition. Previous research has shown the importance of congruency in face identification^56–58^. For example, faces learned with a ski mask are better recognized when they are also presented with a ski mask compared to full-view faces^57^. In our study, there is an incongruence between learning and recognition, as the aperture was only applied during learning (Experiment 1) or recognition (Experiment 2) stages. However, as all our participants were given the same tasks, it is unlikely that incongruence between learning and recognition explains any observed relationships between face recognition skills and the different conditions of the FTAP.

Second, it could be argued that the FTAP also disrupts featural processing. For example, the FTAP might impair the encoding of featural information at the learning stage, which would explain why aperture accuracy was not associated with FRA in Experiment 1. However, this also seems unlikely. Research has shown that holistic processing is mostly engaged by the presence of a whole and intact face^12,59,60^. Importantly, this whole and intact face processing is indeed avoided by the aperture. To ensure the serial processing of each facial feature, the aperture used in this study was created to be large enough to reveal the entire eye and mouth regions, and approximately 75% of the nose. Therefore, although the serial presentation of the features through the aperture might also impair some featural processing, it seems unplausible that this disruption is comparable to that of holistic processing. In fact, if such disruptions were comparable, as observers would not be able to use either featural or holistic processing, performance in the aperture conditions should be at chance levels^61^. However, as our results showed, participants’ performance in the aperture condition was above chance levels in both experiments.

Third, we applied the regression method to compute the holistic advantage of participants. While this approach does control for variance in the aperture condition^14^, one important limitation of the regression method is its assumption of a linear relationship between the whole and aperture conditions. In fact, as shown by the weak correlations, it is possible that a non-linear model could better explain the relationship between the whole and aperture conditions.

In conclusion, we show that poor FRA arises from the poor encoding of holistic and featural information during face recognition. We also show that enhanced holistic (but not featural) processing during face learning contributes to better FRA. In addition, our findings raise the intriguing possibility that good recognisers’ ability to effectively utilise featural information during recognition may depend on the extent to which faces are processed holistically during learning. We demonstrate these using the FTAP that deals with several limitations of other paradigms (i.e., inversion, composite and part-whole tasks). Moreover, the FRA of our sample is broad, to the extent of capturing individuals with FRAs (according to CFMT scores) similar to DPs and SRs identified in past studies, as well as those in between. Therefore, we provide reliable insight into the contribution of holistic and featural processing during face learning and face recognition.

## Methods

### Participants

An *a-priori* power analysis using G*Power^62^ estimated that a sample size of 82 is required to obtain a moderate effect size of 0.3 with a statistical power of 80% (α = 0.05), for a Pearson’s test of correlation between FRA and the conditions of the RMT. In Experiment 1, we recruited 87 Malaysian Chinese (44 females) participants with no known clinical diagnosis of a mental health disorder, with age ranging from 18 to 54 years (*M* = 25.00 years, *SD* = 5.29). For Experiment 2, we recruited 86 healthy typical Malaysian Chinese participants (70 females), with age ranging from 18 to 47 years (*M* = 22.34 years, *SD* = 5.10). Participants were paid 5 Malaysian Ringgits as compensation for their time. All participants reported normal or corrected-to-normal vision. A digital informed consent was obtained prior to participation. All experimental procedures were approved by the Science and Engineering Research Ethics Committee of the University of Nottingham Malaysia (approval code: BLQZ210421). We confirm that all experiments were performed in accordance with relevant guidelines and regulations.

### Apparatus

This study was conducted using the online experimental platform Testable (www.testable.org) ^63^. The study comprised two tasks: the CFMT-Chi^35^ and an old/new recognition memory task (RMT) with two viewing conditions (whole or aperture viewing). Participants used their own computers (laptops or desktops) to complete the two tasks online in a web browser. To minimise differences in the visible size of stimuli across different computer screens, participants were required to adjust the length of a horizontal yellow line that appeared on the screen to match the size of a debit/credit card they possessed. Based on this, the testing platform calculates how many pixels correspond to one centimetre, and all stimuli within the study were rescaled using this mapping to the required dimensions in centimetres. All face stimuli were edited and cropped using Abobe Photoshop CS6, while the dynamic aperture was created in Matlab R2019b (Mathworks).

### Stimuli and procedure

#### Cambridge face memory test-Chinese (CFMT-Chi)

We used the validated Chinese version of the Cambridge Face Memory Test (i.e., CFMT-Chi), and all faces and procedures were the same as those used in the original paper by McKone et al.^35^ Face images were those of men in their 20s and early 30s in neutral expressions, and each individual was photographed in the same range of poses and lighting conditions. For this task, six unique target identities and 46 unique distractor identities were used. For each identity, three face images from three different viewpoints (one left 1/3 profile, one full-frontal and one right 1/3 profile) were used. Similar to the original version, only male faces were used because sex differences in observers have been reported for recognising female but not male faces^64^. These faces did not contain external features, such as hair and no facial blemishes were visible. They were greyscale faces (approximately 160 pixels (px) in width and 195 px in height; assuming participants had a seating distance of 57 cm, the faces subtended approximately 3.2° and 4° in width and height, respectively) embedded in the centre of a uniformly grey background that is 200 px wide and 240 px tall (4 × 4.8 cm; see McKone et al.^35^ for further details).

The CFMT-Chi was presented using the standard procedure which consists of a total of 72 trials presented over three different stages (18 in the Learning, 30 in the Novel and 24 in the Noise stages). In all trials that test face memory, there were three simultaneously presented faces (one learned target and two distractors) and participants were required to select which of them was the learnt face, by pressing the keys “1” for the left, “2” for the middle, “3” for the right image.

#### Old/new recognition memory task (RMT)

Face images were those of Malaysian Chinese males in their early or mid-20s in neutral expressions. All individuals were photographed in the same range of poses and lighting conditions in the Face Laboratory at the University of Nottingham Malaysia, wherein the informed consents to publish identifying information/images were obtained. For each identity, only frontal view face images were used. All external features in the faces were removed. The faces were then resized to approximately 160 px in width and exactly 195 px in height (subtending approximately 3.2° × 4° at a viewing distance of 57 cm), converted into greyscale and embedded in the centre of a uniformly black background of 200 × 250 px (4 × 5 cm).

In Experiment 1, the RMT consisted of four blocks (two whole and two aperture conditions). The four blocks were randomized across participants. Each block started with an initial “learning” stage, followed by a filler task and finally a “recognition” stage. In any given block, the learning stage showed the faces of six unique identities to participants. The recognition stage sequentially presented the same six identities (“old”) randomly intermixed with 6 new and unique identities that the participants had not seen before (“new”), leading to a total of 12 test faces. This led to a total of 48 unique faces (e.g., 24 old and 24 new unique identities) that were used throughout the entire experiment. In the learning stage of the “whole” condition, each trial started with a white central fixation cross (22 × 22 px; 0.4 × 0.4 cm) shown for 500 ms, followed by a fully visible unique face stimulus presented in the centre of the screen for 1000 ms (Fig. 1). Old faces presented in the “whole” condition during the recognition stage are exactly the same as those in the learning stage. In contrast, in the learning stage of the “aperture” condition, the face image was shown through a dynamic window that moved smoothly from the top of the face to the bottom, revealing features of the face in a sequential order (Fig. 1). The dynamic window started and ended with a fully black display. The height of the aperture that moved from top to bottom was 12% (i.e., 30 px) of the overall height of the face and took approximately 6200 ms to move across the entire face (i.e., black-to-black display). The sequential display and frame rate generated a smooth aperture motion (~ 11 frames per second). All sequences were constructed from a series of bitmap images and saved as .GIF files. For both conditions, six of such trials were presented in the learning stage, and participants were asked to learn and memorize all six faces for a subsequent recognition stage.

Following the learning stage, in both conditions, participants were given a short filler task that involved mathematical calculations (e.g., “5 − 6/2 + 10 = ?”), which took less than a minute to complete. This was followed by the recognition stage. During this stage, the 12 test faces were sequentially presented over 12 trials. Each trial began with a 500 ms presentation of a white central fixation cross. This was followed by the presentation of a fully visible face that remained on the centre of the screen until a response was recorded. The participants were required to indicate whether they had previously seen this face in the learning stage, by pressing the key “Q” on the keyboard if they have seen it and the key “P” if they have not seen it before. Participants were instructed to respond as quickly and accurately as possible. In both stages, the presentation timing was adopted from previous studies using the FTAP^26,27^. In the whole condition, old faces presented during recognition and learning were both fully visible. However, in the aperture condition, old faces shown during learning were viewed through an aperture and when the same identities were shown during recognition, they were fully visible.

In Experiment 2, experimental procedures and stimuli used were similar to Experiment 1, except for the following changes in the old/new RMT. Irrespective of the experimental condition (whole or aperture), participants were always shown a white central fixation cross, followed by fully visible faces for 1000ms in the learning stage. A total of six unique faces (i.e., old faces) were shown in each block. During the “recognition stage”, they were shown with the 12 test faces that were either in full-view (for the “whole” condition) or viewed through an aperture (for the “aperture” condition). Faces to be recognised stayed on screen for the same duration of 6200 ms in both conditions, and this was followed by a black screen that remained until a response was recorded. Responses could also be provided while the faces were shown or after the faces were removed from the screen, either of which terminated the trial. Similar to Experiment 1, participants pressed the key “Q” or “P” to indicate whether they have seen each test face in the learning stage or not, respectively.

### Supplementary Information

Supplementary Information.

## Data Availability

The datasets generated during and/or analysed during the current study are available in the Open Science Framework repository, https://osf.io/a7evh/?view_only=456b591ea31c472f8118bbdeddb1b5de.
