# Supplementary material for: Holistic and featural processing’s link to face recognition varies by individual and task
Source: Sci Rep. 2023 Oct 6;13:16869. doi: 10.1038/s41598-023-44164-w (PMC10558561; doi:10.1038/s41598-023-44164-w)
Supplement: Supplementary file 1 — Supplementary Information. [file 41598_2023_44164_MOESM1_ESM.pdf]

Online Supplementary Material

Although the overall aperture condition accuracies were above chance at the group level, the associations (or the lack of them) between aperture accuracies/RAE scores and CFMT-Chi could be driven by *near or below chance* performances in some participants (i.e., floor effects). To address this concern, we identified individuals who did not perform above chance levels and removed them from the correlation tests. For this purpose, we used the binomial distribution and calculated the accuracy required to reject the null hypothesis that accuracy was simply based on guessing. Given that we had 24 trials per condition, with a chance-level performance set at 0.5, we estimated that above chance performance is characterised 17 or more correct trials per condition. The cumulative binomial probability that this accuracy (or higher) occurs if the null hypothesis is true is 0.032, which is less than the significance criterion of $\alpha = 0.05$. Accordingly, for the correlations between aperture accuracy and CFMT-Chi, we only included participants with 17 or more correct trials (16 and 18 participants for Experiment 1 and 2, respectively), and the same for the correlation between whole accuracy and RAE scores with CFMT-Chi (41 and 60 participants for Experiment 1 and 2, respectively).

For Experiment 1, there was a significant positive correlation between the whole accuracy and CFMT-Chi, $r(39) = .380$, $p = .014$, but not between aperture accuracy and CFMT-Chi, $r(14) = -.363$, $p = .167$. There was also a significant positive correlation between the RAE scores and CFMT-Chi scores, $r(39) = .325$, $p = .038$. For Experiment 2, we found significant positive correlations between the accuracy in the whole condition and participants' respective scores on the CFMT-Chi, $r(58) = .327$, $p = .011$, accuracy in the aperture condition and their respective scores on the CFMT-Chi, $r(16) = .534$, $p = .022$, as well as between the RAE scores and CFMT-Chi scores, $r(58) = .289$, $p = .025$. While the pattern of results remains similar to

what we report in the main manuscript, the significance levels of the correlations we report here are relatively lower. This most likely reflects the exclusion of a significant number of participants from the main analysis. Overall, these findings further confirm our interpretations that the difference in associations between featural processing and face recognition ability during different stages of face recognition is not due to floor effects.
